# Remarks on the Influence of the Weather on Man, and on the Use of Sea-Bathing

**Published:** 1836-10

**Authors:** 


Art. XI.—*Einige Bemerkungen über den einfluss der Witterung auf den Menschlichen organismus überhaupt, und insbesondere auf die anwendung der Seebäder in Dobberan.* Vom Dr. Johann H. Becker.—*Parchim,* 1835. 8vo. pp. 89.

*Remarks on the Influence of the Weather on Man, and on the Use of Sea-bathing.* By J. H. Becker, m.d.—*Parchim,* 1835.

Dobberan is a sea-bathing town in Mecklenburg, on the southern shore of the Baltic, nearly opposite the island of Zealand, and Dr. Becker is a physician connected with the bathing establishment of the place. As his pamphlet, like many that emanate from the press of watering places of all countries, is calculated—and probably intended—rather to enhance the local renown of the residence and the doctor, than to add to the stock of medical knowledge, we shall pass over the main subject of it entirely, and only notice one thing which we consider as both interesting and important, and which we find here treated of more fully than in any other work to which we can now refer. This is the *temperature of the sea,* and the relation of this to the temperature of the atmosphere. It certainly is rather odd that we, in this sea-land of ours, with our one encircling coast and our thousand towns built on the very verge of the ocean, should have to go to an obscure town in Mecklenburg and to a German doctor for information on such a subject. But there is no limit to the enthusiasm and laborious industry of the Germans.

Independently of the scientific interest attaching to it, the temperature of the sea on coasts is of much more importance, in a medical point of view, to those who use sea-bathing, than is generally imagined; and we are quite sure that, if more attention were paid to this matter by the medical residents at our sea-bathing places, they would not only be better able to explain the anomalies in the effects of the sea-bath, so frequently witnessed by them, but would also be capable of laying down much more precise rules for the proper seasons and times of bathing: and, if we possessed proper registers of the summer temperature of the sea on our different coasts, we should be much better able than at present to fix on the best resort for particular invalids. The attention of the profession is now much alive to the difference of *atmospheric* temperature in different localities of our island, and this is universally considered in making choice of a residence for invalids in the winter season. In sending patients for sea-bathing, however, the temperature of the sea water is never considered; and yet the fact is, that this varies very considerably in different places; much less, indeed, than that of the air, yet sufficiently to make the difference a cause, in certain delicate subjects, for preferring one bathing place to another. The most influential source of the difference of temperature on our coasts is their relative steepness or boldness, flatness or shallowness. On a bold rocky coast, where the ebbing of the sea is shown rather by a perpendicular than a horizontal recession of the water, there will be much less difference of temperature in the water at different days, and at different hours in the same day, than on a low flat shore, from which the tide recedes to a great distance. In the latter case, the warm summer sun will exert a much greater heating power over the comparatively thin stratum of water spread over the flat ground, than on the deep waters of the bold coast; and, more particularly, if the ebb takes place in the forenoon, leaving the low, sandy, or shingly beach exposed to the sun's rays during the hottest part of the day, the flowing tide of the afternoon will not only receive its complement of heat from the direct rays of the sun, but will take up that which had been imparted to the expanded shore exposed during the ebb. The consequence of this is, that the temperature of the sea-water on a shallow coast is often not only as high, but actually higher, than that of the air; and, when it is further considered that cold weather produces analogous but opposite results, the shallow water being as readily cooled as it is warmed, we shall be convinced that this is really a matter of importance. We know, by experience, that the sea-water at one of our bathing places varies in the course of the same season, at different days and hours, upwards of twenty degrees; and in Dr. Becker's tables we observe a difference of twenty-three degrees; and, although a much less difference of temperature than this is justly regarded as of great importance, and is always attended to in prescribing an ordinary bath for our patients, we never think of it in prescribing a bath in the open sea.

As in the Baltic sea there is very little ebb or flow, the sources of variation of temperature in the water of the coasts are fewer; but, as the shores of that sea are generally low and flat, (at least the southern, the water is much influenced by the sun's rays and the atmospheric temperature. The tables of Dr. Becker, therefore, will not be considered as offering any close approximation to the results likely to be obtained on the coasts of this island. Still they are interesting and important, and afford many valuable analogies which directly apply to our own seas.

In one table, Dr. Becker gives us the maximum and minimum monthly temperature of the sea at Dobberan, at seven a. m. and four p. m. for the months of June, July, August, and September, for a period of nineteen years, viz. from 1818 to 1832 inclusive, (with the exception of the year 1829); in two others, he gives us the daily temperature of the sea and the air, at the same hours, during the same months of the years 1833 and 1834. From these we have extracted the following general results, which will convey a more precise idea of the phenomena than the full details of the original tables. We will only premise, that the temperature was always taken at the same spot, where the depth of the water is from seven to eight feet, the thermometer (Fahrenheit's) being sunk to the bottom, and kept from five to eight minutes in that position.

TABLE I. *Temperature of the Sea at Dobberan,* (*Results of nineteen Years,* 1813-1832.)

| MONTH. | June. | | July. | | August. | | Septemb. | |
|---|---|---|---|---|---|---|---|---|
| Hour of Observation. | At 7 A.M. | At 4 P.M. | At 7 A.M. | At 4 P.M. | At 7 A.M. | At 4 P.M. | At 7 A.M. | At 4 P.M. |
| Absolute maximum ...... ............ | 66 | 75 | 63 | 76 | 69 | 75 | 68 | 72 |
| Absolute minimum ............ ...... | 50 | 52 | 54 | 56 | 57 | 58 | 53 | 54 |
| Mean of the maxima .............. | 62.6 | 66.6 | 66.4 | 71.3 | 65.5 | 69.8 | 61.7 | 61.8 |
| Mean of the minima.................. | 54.5 | 56.5 | 57.9 | 60.5 | 60.1 | 61.3 | 55.2 | 56.8 |
| Mean of the maxima and minima | 58.5 | 61.5 | 62.1 | 65.9 | 62.8 | 65.5 | 58.4 | 59.3 |
| Mean daily temp. of each month, calculated from both observations. | 60 | | 64 | | 64.1 | | 58.8 | |

TABLE II. *Relative Temperature of the Sea and Air during* 1833 *and* 1834.

| | | June. | | | | July. | | | | August. | | | | September. | | | |
|---|---|---|---|---|---|---|---|---|---|---|---|---|---|---|---|---|---|
| | | 7 A.M. | | 4 P.M. | | 7 A.M. | | 4 P.M. | | 7 A.M. | | 4 P.M. | | 7 A.M. | | 4 P.M. | |
| | | Sea. | Air. | Sea. | Air. | Sea. | Air. | Sea. | Air. | Sea. | Air. | Sea. | Air. | Sea. | Air. | Sea. | Air. |
| Max. | 1833 | 60 | 63 | 64 | 80 | 63 | 64 | 67 | 69 | 62 | 59 | 66 | 65 | 58 | 58 | 60 | 62 |
| | 1834 | 64 | 66 | 68 | 79 | 70 | 72 | 74 | 90 | 70 | 70 | 75 | 79 | 69 | 67 | 70 | 78 |
| Min. | 1833 | 52 | 54 | 58 | 57 | 59 | 55 | 60 | 55 | 55 | 50 | 58 | 55 | 54 | 49 | 55 | 50 |
| | 1834 | 54 | 50 | 56 | 54 | 60 | 58 | 63 | 64 | 63 | 52 | 66 | 68 | 52 | 50 | 58 | 57 |
| Med. | 1833 | 57.4 | 58.5 | 59.7 | 62.4 | 60.2 | 59.2 | 63.3 | 63.4 | 57.9 | 54.6 | 60.2 | 59.6 | 56.1 | 54 | 58.1 | 57.8 |
| | 1834 | 59.4 | 58 | 63.2 | 64.3 | 65.3 | 64.9 | 68.9 | 71.6 | 68.4 | 64.3 | 70.1 | 71.1 | 65.4 | 57.3 | 63.8 | 65.8 |

These tables clearly demonstrate the complete dependence of the sea on the air for its temperature, at least at Dobberan. Although we find considerable discrepancies, as might be expected, in the relation of the maxima and minima of the air and water to each other, in the different times of the day and in the different months, still it will be seen that the relation of the general or mean temperature of these coincide very closely. Thus, we find, from the first table, that the water is in all the months warmer in the afternoon than in the morning, and warmer in the months of July and August; than in the preceding or subsequent months; and we know that this is the case with the temperature of the air. But the connexion between the temperature of the two media is still more strikingly shewn in the second table, where we see the mean temperature of both rising and sinking together, at the different hours of observations, through the different months, and in the whole season. To notice only one proof of this fact: the two summers, 1833 and 1834, differed greatly in their temperature, the latter being much the warmest of the two, and the evidence of this is quite as conspicuous in the temperature of the water as in that of the air.

This will appear very striking from the following results deduced from that table.

| | 1833. | | 1834. | |
|---|---|---|---|---|
| | Air. | Sea. | Air. | Sea. |
| Mean of the maxima ...... ........ | 65 | 62.4 | 74.7 | 70 |
| General mean temperature ...... | 58.6 | 59.1 | 64.6 | 65.5 |

Here we observe that, whilst there is a very great difference between the actual temperature of the two years, there is a very slight difference between the relative temperature of the sea and air in both.

